# Calcitonin native prefibrillar oligomers but not monomers induce membrane damage that triggers NMDA-mediated Ca^2+^-influx, LTP impairment and neurotoxicity

**DOI:** 10.1038/s41598-019-41462-0

**Published:** 2019-03-26

**Authors:** Marcello Belfiore, Ida Cariati, Andrea Matteucci, Lucia Gaddini, Gianfranco Macchia, Raoul Fioravanti, Claudio Frank, Virginia Tancredi, Giovanna D’Arcangelo, Marco Diociaiuti

**Affiliations:** 1 National Center for Rare Diseases, Istituto Superiore di Sanità, Rome, Italy; 2 Department of Systems Medicine, University of Rome Tor Vergata, Rome, Italy; 3 National Center for Drug Research and Evalutation, Istituto Superiore di Sanità, Rome, Italy; 4 Core Facilities Service, Istituto Superiore di Sanità, Rome, Italy; 5 Chemistry Department, University “Sapienza”, Rome, Italy

## Abstract

Amyloid protein misfolding results in a self-assembling aggregation process, characterized by the formation of typical aggregates. The attention is focused on pre-fibrillar oligomers (PFOs), formed in the early stages and supposed to be neurotoxic. PFOs structure may change due to their instability and different experimental protocols. Consequently, it is difficult to ascertain which aggregation species are actually neurotoxic. We used salmon Calcitonin (sCT) as an amyloid model whose slow aggregation rate allowed to prepare stable samples without photochemical cross-linking. Intracellular Ca^2+^ rise plays a fundamental role in amyloid protein-induced neurodegerations. Two paradigms have been explored: (i) the “membrane permeabilization” due to the formation of amyloid pores or other types of membrane damage; (ii) “receptor-mediated” modulation of Ca^2+^ channels. In the present paper, we tested the effects of native sCT PFOs- with respect to Monomer-enriched solutions in neurons characterized by an increasing degree of differentiation, in terms of -Ca^2+^-influx, cellular viability, -Long-Term Potentiation impairment, Post-Synaptic Densities and synaptophysin expression. Results indicated that PFOs-, but not Monomer-enriched solutions, induced abnormal -Ca^2+^-influx, which could only in part be ascribed to NMDAR activation. Thus, we propose an innovative neurotoxicity mechanism for amyloid proteins where “membrane permeabilization” and “receptor-mediated” paradigms coexist.

## Introduction

Protein misfolding is implicated in several severe amyloid-related neurodegenerations such as Alzheimer’s (AD), Parkinson’s, and Creutzeld-Jacob’s diseases and type 2 diabetes mellitus. In these conditions, the first step in the amyloid formation pathway is the destabilization of the native core protein conformation. Proteins associated to the various neurodegenerative diseases, including Amyloid-β (Aβ), human islet amyloid polypeptide, α-synuclein peptide and prion protein, display the tendency to self-assemble in the amyloid pattern. Despite they do not share sequence homologies, the process is similar and results in a self-assembling behaviour leading to the formation of soluble oligomers that share morphology and dimensions^1^. Notably, as suggested by Glabe, the fact that amyloids share generic structures implies that they may also share a common pahological function^2^.

Briefly, in the early stages of this process a dynamic equilibrium between small pre-fibrillar oligomers (PFOs), proto-fibrils (PFs) and mature and insoluble mature-fibres (MFs), is achieved. As the process moves forward, random coiled structures (PFOs) interchange into β-sheet conformation (PFs and MFs)^3^. Before the beginning of the PFs formation, the aggregation process is quite complicated since different metastable structures can be formed from Monomers. Several off-pathways exist, resulting in intermediates like random coiled non-fibrillating aggregates, metastable β-sheet intermediates and α-helix micelles^4^. All these structures are named PFOs and are common to many amyloid proteins (for details see Diociaiuti *et al*.^3^). This process happens during a period of time named “lag-phase”, whose duration depends on protein concentration, ionic strength, pH and temperature. Moreover, also the protein sequence strongly affects the duration of the “lag-phase” and aminoacid substitutions can change the amyloidogenic property of a protein^4^. Upon formation of a critical aggregating β-sheet core, aggregation is auto determined and the elongation proceeds fast.

Although extensive studies over the last decades have been made, many details regarding the role played by PFOs in the early stages of neurotoxicity have not been elucidated yet and which is the most neurotoxic species among oligomers is still debated. Many different culprits have been proposed for Aβ toxicity: Monomers, SDS-stabilized dimers, 56 KDa aggregates, diffusible ligands arising from βA, LPFs, APFs and MFs. However, due to their intrinsic instability and the different protocols of sample preparation, aggregate structures may change during experiments making the interpretation of data very complicated. As reviewed by Benilova *et al*. for Aβ, the lack of a common, agreed-upon experimental description of the toxic oligomer, makes interpretation and direct comparison of data between different research groups very difficult^5^. Anyway, it is now generally accepted that small soluble oligomers are more toxic than MFs, which are considered almost inactive or even protective, due their capacity to subtract soluble toxic species from the equilibrium^5^.

At the present, many efforts are focused to clarify the relationship between oligomers structures and cytotoxicity, based on analysis of cytotoxic effects *in vitro*, on neuronal cultures and brain slices and *in vivo*, by direct administration to the brain^6^. To overcome the difficulties discussed before Bitan and Teplow developed a methodology named Photo Induced Crosslinking of Unmodified Proteins (PICUP)^7^ and other groups demonstrated that this method allows isolation of cross-linked Monomers, dimers, trimers and tetramers^8^. This rise the possibility to test them separately on biological systems. However, we can speculate that, during PICUP, the formation of new bonds could change biological effects of these molecules, potentially inducing a bias in the analysis of their toxicity or in the molecular mechanism of action. Recently, novel approaches in the study of the toxicity of unfixed assemblies, *in vitro* and *in vivo*, have been proposed by Beeg *et al*. based on Surface Plasmon Resonance thecnique and the use of *Caenorhabditis elegans*^9^.

Calcitonin (CT) is a 32 aminoacid polypeptide hormone secreted in the ultimo-pharyngeal body of the fish and in thyroid of other vertebrates, including mammals^4,10^. This protein has a pivotal role in the maintenance of blood Ca^2+^ and bone homeostasis. CT displays the tendency to self-assemble in the amyloid pattern even if this behaviour does not lead to a neurodegenerative disease^4^. Among CT variants, salmon Calcitonin (sCT) presents the lowest aggregation rate^4^. While human CT (hCT) lag-phase is 21 hours (1 mg/mL at 22 °C in 50 mM Tris/HCl buffer, pH 7.4), in the sCT under the same conditions is above 8 months^4^. Due to this appealing property, we successfully used sCT as a tool to investigate the effects of native PFOs in the early stages of neurotoxicity without the need of stabilization procedures such as Photo-induced Cross-linking of Unmodified Proteins (PICUP)^7,11^. In previous biophysical investigations we demonstrated that native sCT PFOs formed oligomeric pore-like structures in artificial liposome membranes^12^, and that the presence of monsialoganglioside-GM1 (GM1) triggered binding of sCT to a Langmuir membrane model mimicking lipid-raft composition^13^. Furthermore, we showed that sCT was strongly neurotoxic and that its neurotoxicity was related to the composition of neuronal membrane^14^. Then, we prepared by size exclusion chromatography (SEC) sCT PFOs enriched fractions and we demonstrated that they were neurotoxic^3^.

It has been generally accepted that in amyloid neurodegenerative diseases, neurotoxicity and neurodegeneration start early, but symptoms are seen later on^15^. This common feature prompted researcher to investigate time-depending effects. Several acute amyloid effects have been analysed in terms of second messengers transients^16–19^ or signalling pathways activation^16,20,21^, while delayed effects have been analysed investigating long-term effects like apoptosis^22,23^, cellular viability, neuronal fibre loss, synaptic plasticity impairments^6,24^ and oxidative stress^25^. Post-synaptic densities (PSD) proteins are part of a complex cluster located in the postsynaptic regions of dendritic spines. It has been suggested that these proteins may contribute to synaptic plasticity by regulating trafficking and localization of glutamatergic receptors within the PSD, playing a critical role in neuronal activity^26^. The PSD-95, a membrane-associated guanylate kinase, is the most important scaffolding protein in the PSD. It is involved in the binding of many receptors, such as N-methyl D-aspartate receptors (NMDARs)^27,28^ and α-amino-3-hydroxy-5-methyl-4-isoxazolepropionic acid receptor (AMPARs) via Stargazin/TARP proteins, thus playing a pivotal role in the modulation of synaptic strength^29–36^.

As reviewed by Angelova and Abramov, it is now hypothesized that the rising of intracellular Ca^2+^ represents a common mechanism in several neurodegenerative diseases that involves mitochondrial dysfunction and oxidative damage, leading to cell death^37^. However, the molecular mechanisms and in particular the determinants at the base of the Ca^2+^ dyshomeostasis in neurons, are not clear yet.

Up to now, two paradigms can be found in the literature to explain the observed Ca^2+^-influx and the consequent biological effects:

(i) The “membrane permeabilization” hypothesis, where the alteration of the neuron membrane, due to the formation of pore-like structures^17,38,39^ or to the incorporation of oligomers in the lipid bilayer, induces membrane thinning, reduction in the dielectric barrier and increase in membrane permeability^40,41^. Recently, Bode and colleagues demonstrated ion-channel formation induced by Aβ-42 oligomers but not by SEC-purified Monomers in cellular membranes excised from HEK293 cell line^42^. Furthermore, based on transmission electron microscopy and atomic force microscopy studies, Shafrir and co-workers simulated, by Molecular Dynamics, the formation of stable amyloid pores^43^. It has been also proposed that the entry of small molecules and ions, such as Ca^2+^, can reflects in acute excitotoxicity^44^. It’s now accepted that lipid-rafts play a pivotal role in the interaction between amyloid oligomers and membranes, as reported in our paper concerning the binding of sCT to a Langmuir membrane model^13^. More in particular, GM1 seems to be a specific target of amyloids. Hong *et al*. reported that Aβ dimers strongly reduced Long-Term Potentiation (LTP) in mouse hippocampal slices, and this impairment was due to the interaction with membranes mediated by GM1. They also showed that a pre-treatment with Cholera Toxin Subunit β (CTβ), which masks the two terminal sugars of GM1, interfered with this binding and abolishes the LTP depression^24^.
(ii) The “receptor-mediated” hypothesis, where amyloid oligomer modulation of Ca^2+^ channels such as NMDAR, AMPAR or voltage-dependent (V-dependent) channels, leads to an abnormal intracellular Ca^2+^ concentration^5,45^. Several groups reported on the relationship between Aβ oligomers and NMDA excitotoxicity^20,46^. Peters *et al*. demonstrated that Aβ-evoked ion currents in hippocampal neurons are proportional to neuronal NMDAR expression^47^. It has been also reported that amyloid oligomers can elevate intracellular Ca^2+^, through a signalling pathway depending upon specific Ca^2+^/calmodulin protein phosphatases present at PSD^45,48^. Other groups demonstrated that, in hippocampal neurons, Aβ oligomers activate metabotropic glutamate receptors-5 (mGluR5), resulting in the activation of JNK, Cdk5 and p38 MAPK pathways^31,45,49,50^. Moreover, it has been also suggested that LTP inhibition would be a consequence of Aβ-oligomers interference with glutamate synaptic reuptake mechanism^30^. Notably, it is well known that the Ca^2+^ channel occurrence is correlated to the lipid-rafts and then to the GM1 expression.

In the present paper, we used sCT as a tool to investigate the molecular mechanisms of amyloid neurotoxicity in neuronal systems characterized by increasing occurrence of lipid-rafts, marked by GM1. Firstly, in neuronal cultures characterized by increasing degree of differentiation (HT 22, HT-22 DIFF, HpC 6 DIV, HpC 14 DIV) we tested the effects of native sCT PFOs- with respect to Monomer-enriched solutions, in terms of intracellular Ca^2+^-influx, reduced cellular viability and PSD-95 expression. Finally, we assessed LTP impairment in mouse hippocampal slices.

Our results show that native metastable sCT PFOs induced biological effects whose intensity increased with the differentiation degree of neuronal cultures, while Monomers were always ineffective. Notably, we propose for the first time to our knowledge, a possible mechanism of action where both “membrane permeabilization” and “receptor-mediated” paradigms could contribute to explain the experimentally observed intracellular Ca^2+^-influx. We speculate that in mature neurons PFOs, but not Monomers, may induce an initial membrane damage that triggers a subsequent abnormal NMDA-mediated Ca^2+^-influx, leading to the observed LTP impairment and neurotoxicity.

## Results

### Sample preparation and characterization

Native sCT solution was prepared by incubating 2 mg of sCT powder in phosphate buffer (5 mM) at room temperature overnight. In a previous paper^3^, we showed that, under these conditions, the formation of PFOs was favoured with respect to MFs. In order to purify oligomeric species enriched fractions, the aggregated and native solution was then loaded in the SEC column (detailed procedures of SEC are reported by Diociauti *et al*.^3^).

**Figure 1:**
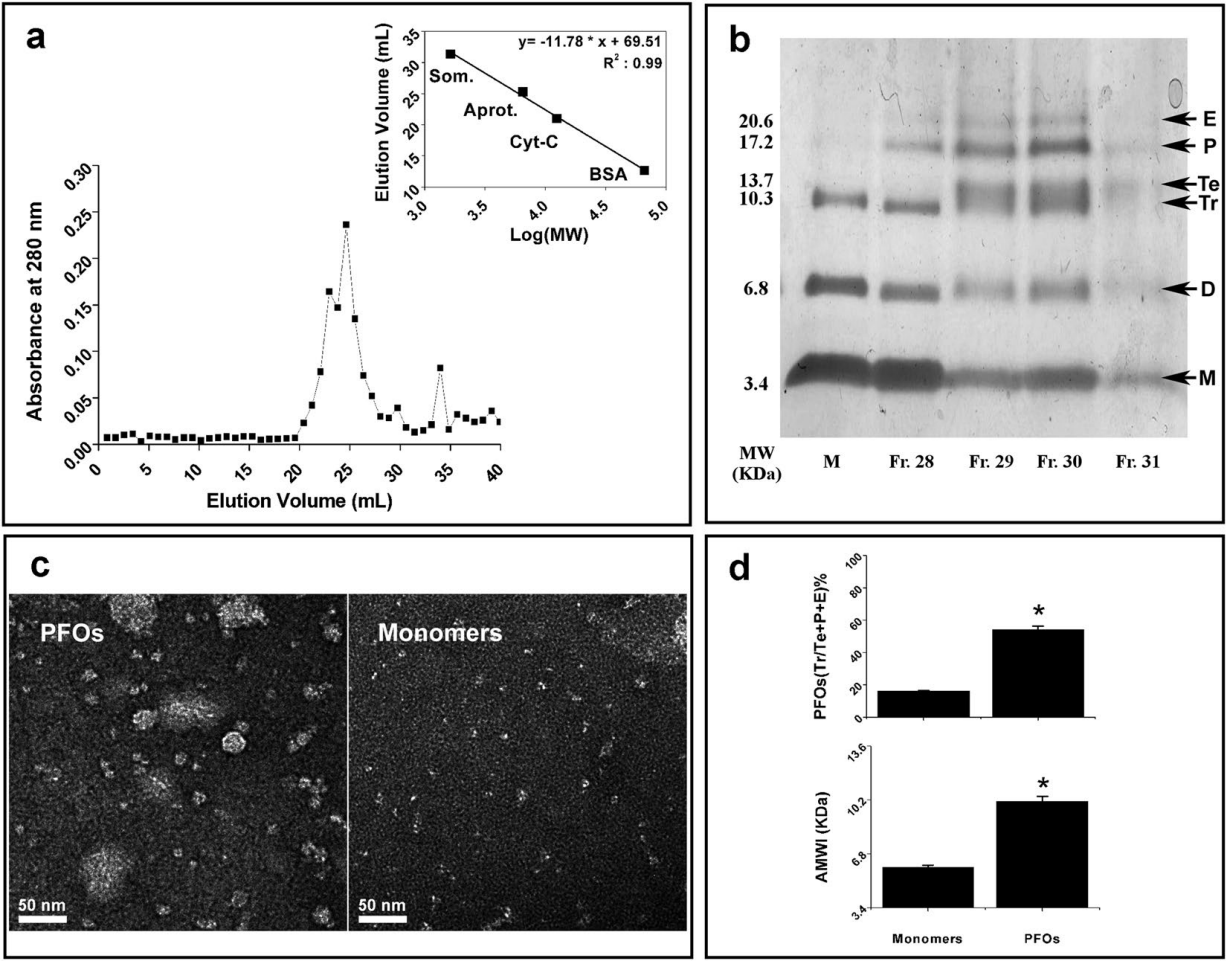
The samples and their characterization. (**a**) Shows the Size Exclusion Chromatography elution profile of the spontaneously aggregated sCT sample. A main peak, relative to PFOs, can be observed around 29^th^ fraction corresponding to V_e_ = 24.65 mL. The calibration regression (inset) gives MW = 6450 Da, which is about a sCT dimer. (**b**) shows the gel characterization relative to 28, 29, 30 and 31^th^ fractions (V_e_ = 23.80, 24.65, 25.50 and 26.35 mL), enriched in trimers, tetramers, pentamers and hexamers, compared to the sample rich in Monomers, dimers and trimers. (**c**) Shows high-resolution EF-TEM images relative to PFOs (29^th^ fraction) and Monomers (Bar 50 nm). (**d**) shows the Average-Molecular- and PFO-indexes for the same samples (wiskers indicate SE).

The SEC profile of sCT spontaneously aggregated mixture is depicted in Fig. 1a. The absorbance at 280 nm, along SEC fractions, is characterized by a peak located around 29^th^ fraction (V_e_ = 24.65 mL), followed by a prolonged tail. According to calibration standards elution profile (inset of Fig. 1a), the peak (6450 Da) is equivalent to a dimer.

The tail on the right is due to Monomers that are uniformly distributed in several fractions at a very low concentration. For this reason, we decided to prepare Monomer-enriched sCT solution by dissolving the lyophilized powder in desalted water just before treatments, at concentration of 80 μM, similar to the concentration of the 29^th^ fraction. This because it is well known that the aggregation dynamics depends from ionic strength and concentration.

A characterization of the PFOs fractions, from the SEC peak and Monomers native samples, has been obtained after PICUP procedure followed by tricine SDS-PAGE (Fig. 1b). In the case of PFOs (fractions 29^th^ and 30^th^), the gel analysis clearly demonstrated an upwards-shifted pattern as compared to Monomers sample, where dimers and trimers were also present. A strong enhancement of the aggregated species, such as tetramers, pentamers and hexamers, was observed. To better analyse this trend, we calculated two indexes: (i) the *Average Molecular Weight index* (*AMWi*); (ii) *trimers* + *tetramers* + *pentamers* + *hexamers percentage (PFOs%)* for PFOs-enriched fractions and Monomer unfractioned samples (for details see Fig. S1). Results relative to the 29^th^ and 30^th^ fractions (V_e_ = 24.65 and 25.50 mL), highlighted in Fig. 1d, gave *AMWi* = *1*0*.0 KDa* for sCT PFOs, which is close to trimer molecular weights (10.2 KDa), while *AMWi* = *5.8 KDa* for native unfractioned Monomers, which is between Monomer and dimer molecular weights (3.4 KDa and 6.8 KDa, respectively). The percentages of trimers, tetramers, pentamers and hexamers rises from about 16.5% for Monomer- to 54.0% for PFOs-enriched samples. We noticed that, even if monomeric species were overexpressed in the Monomer-enriched samples, dimers and trimers were always present (Fig. 1b).

We want to stress that, using sCT we did not obtain aggregates of molecular weight higher than hexamers. As reported earlier, unlike others members of the amyloid family, sCT presents a very low aggregation rate with the longest aggregation lag-phase among CT variants^4^. This rises the possibility to produce stable PFOs-enriched samples made of known aggregates to be tested directly onto cells.

Figure 1c shows representative Energy Filtered-Transmission Electron Microscopy (EF-TEM) micrographs relative to the central fraction (29^th^ fraction) of the PFOs peak, together with images relative to the Monomer-enriched sample. Notably, PFOs appeared as particles of 10 ± 3 nm, not perfectly spherical and sometimes characterized by well-defined or incomplete hexagonal shape. Few bigger particles were also present. Conversely, image of Monomer-enriched sample revealed the presence of very small dots of about 3 ± 1 nm, likely Monomers, together with clusters of few dots, dimers and trimers (means of maximum diameters of more than 30 particles per sample type).

## PFOs Administration to Cellular Cultures

### Intracellular Ca^2+^-influx

**Figure 2:**
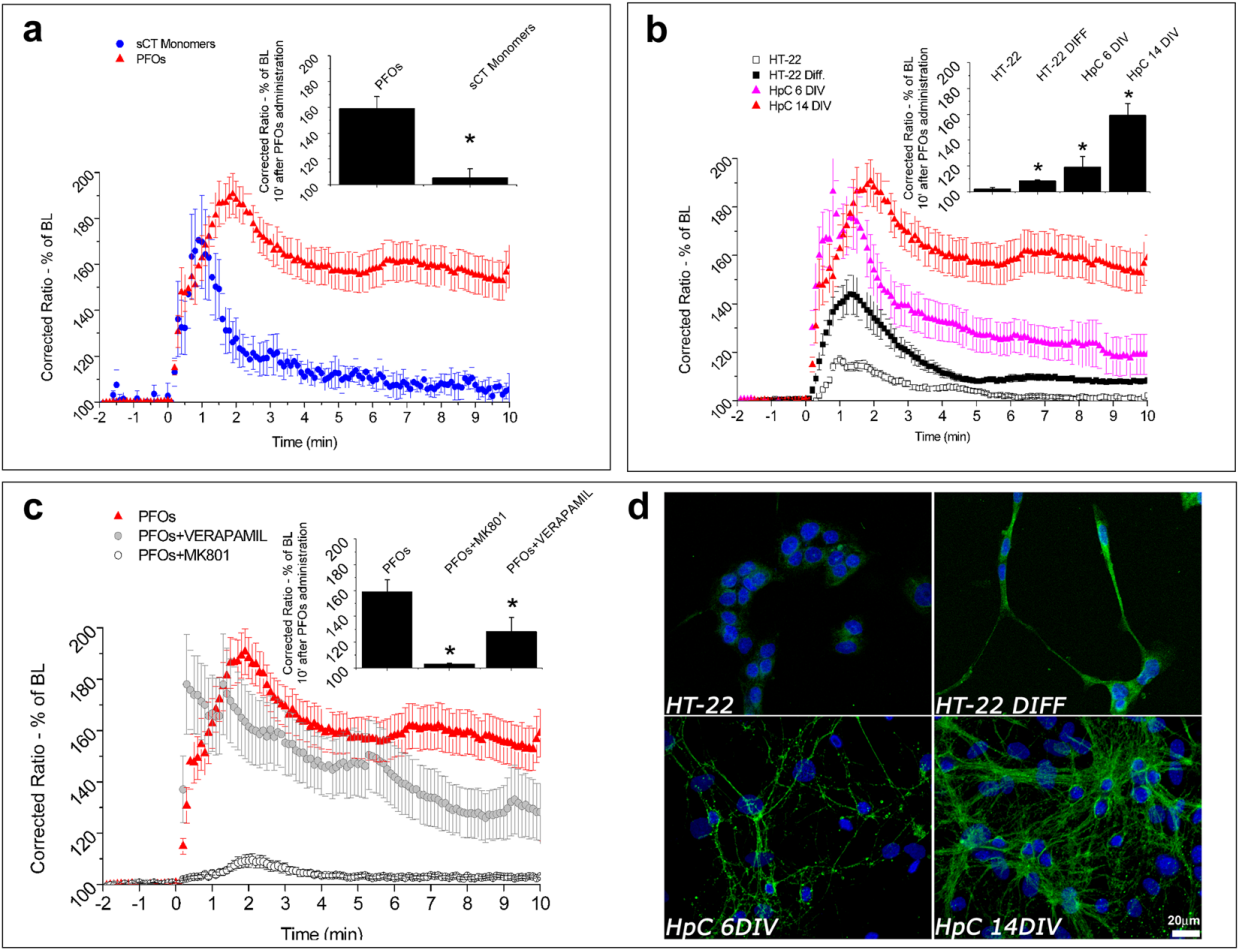
The intracellular Ca^2+^-influx spectra. (**a**) shows the Ca^2+^-influx induced, in 14 DIV primary hippocampal neurons, by sCT PFOs- (n = 76 cells) and Monomer-enriched solutions (n = 23 cells). In (**b**) the differences in the Ca^2+^-influx induced by PFOs in 14 DIV (n = 76 cell), 6 DIV (n = 73 cells), HT22 DIFF (n = 140 cells) and HT22 (n = 659 cells), are reported. (**c**) shows the effects of MK801 (n = 27 cells) and Verapamil (n = 67 cells) on Ca^2+^-influx induced by PFOs in 14 DIV primary hippocampal neurons (insets show values reached after 10 min). (**d**) shows fluorescence microscopy images of the GM1 distribution (green) in the different cell types of increasing differentiation degree (Bar 20 µm).

Here (Fig. 2a) we compared the effects induced by sCT PFOs- and Monomer- enriched sampes on internal [Ca^2+^]_i_ in 14 DIV hippocampal neurons. PFOs enriched fractions induced an elevated [Ca^2+^]_i_ rise, which was maintained after 10 minutes, while sCT Monomers resulted in a fast transient of Ca^2+^-influx peaking in a minute, coming back to the base line after 10 minutes.

In a previous paper, we reported that the Ca^2+^-influx was evoked by not-fractioned mixture of sCT oligomers in 14 DIV hippocampal neurons, which was abolished by disrupting GM1 sialic acid heads by neuraminidase^14^. Thus, we hypothesize that growing levels of this ganglioside would result in the [Ca^2+^]_i_ increase.

To investigate if the Ca^2+^-influx depends on the GM1 content, we compared undifferentiated HT22 cells, differentiated HT22 cells, primary hippocampal neuronal cultures from rat hippocampi at 6 DIV and 14 DIV. In fact, it is well known that the GM1 content increases with neuronal differentiation degree. We verified this fact in our cells by immunofluorescence (Fig. 2d).

Results are reported in Fig. 2b. Significant differences were obtained between all groups. As it can be observed, shape and intensity of the curves changed with the differentiation degree. The high and sustained Ca^2+^-influx of 14 DIV hippocampal neurons was reduced for 6 DIV neurons. The shape was maintained in the first minutes but the plateau reached in the second part was more than halved. HT22 DIFF cells gave rise to less intense signals, with a shape very similar to that of 6 DIV neurons. Finally, we note that undifferentiated HT22 cells showed the smallest Ca^2+^-influx, vanishing after about 6 minutes.

Summarizing, we noted a strong correlation between [Ca^2+^]_i_ signal maintained after 10 ninutes and cell differentiation degree (inset of Fig. 2b).

To investigate the nature of the Ca^2+^-influx we pre-treated 14 DIV neurons with MK801, a specific blocker of NMDAR and with Verapamil, a blocker of V-dependent channels. Results (Fig. 2c) indicated that, after treatment with MK801, the [Ca^2+^]_i_ increase was reduced to a small and transitory peak and this observation suggests that the strong and maintained Ca^2+^-influx of the 14 DIV neurons was due to the NMDARs. Conversely, we found a slight dependence on voltage-activated Ca^2+^ channels, since their specific blocker (Verapamil) determined only a small decrease of the PFOs-evoked response (Fig. 2c).

We want to stress the similarity in shape and intensity between the peak obtained with undifferentiated HT22 cells (Fig. 2b) and the one observed with 14 DIV neurons in the presence of MK801 (Fig. 2c) where NMDARs were inhibited. This similarity suggests that a mechanism different from NMDAR activation was in part responsible for [Ca^2+^]_i_ increase. Notably, as pointed out by He *et al*., these channels were totally missing in the HT22 cells^51^. In order to verify this important issue, we treated both HT22 and HT22 DIFF with NMDA, monitoring the induced Ca^2+^-influx. Results (Fig. S2) clearly showed that Ca^2+^-influx was totally absent in HT22 cells, confirming the lack of NMDARs and demonstrating that the small peak induced by sCT PFOs must be due to a different mechanism.

NMDA induced a gradual raising of [Ca^2+^]_i_ in HT22 DIFF, demonstrating that, as reported by He *et al*., the differentiation process leads to the expression of the NMDARs^51^. However, the shape of the Ca^2+^-influx induced by NMDA in HT22 DIFF (Fig. S2) was very different from that induced by sCT PFOs, lacking the transitory features occurring in the first minutes and characterized by a plateau value reached after 10 minutes, of the same intensity of the plateau reached with sCT PFOs (Fig. 2b). This suggests that NMDARs were responsible for the Ca^2+^-influx obtained at the steady state, in agreement with the conclusion drawn in the case of 14 DIV neurons pre-treated with MK801.

Finally, to investigate the possible correlation between Ca^2+^-influx and GM1 expression, we examined by fluorescence microscopy the membrane distribution of GM1 in all cell types, by using fluorescence-labelled CTβ, known to selectively bind the GM1 sialic acid heads. In HT22 cells, images showed the characteristic dotted distribution and the increase of GM1 expression due to differentiation (Fig. 2d, upper panel) while, in hippocampal cultures, GM1 was evenly distributed throughout the plasma membranes (Fig. 2d, lower panel) and the GM1 content further increased upon differentiation (lower panel). We conclude that a correlation exists between Ca^2+^-influx and GM1 expression.

Summarizing, we provide evidences that sCT PFOs evoked strong and sustained intracellular Ca^2+^-influx mediated by NMDARs and that this behaviour was correlated with the GM1 expression. In the absence of NMDARs or with these channels blocked, and with few GM1 (undifferentiated HT22) a small but detectable Ca^2+^-influx was still observed.

### Cell viability

**Figure 3:**
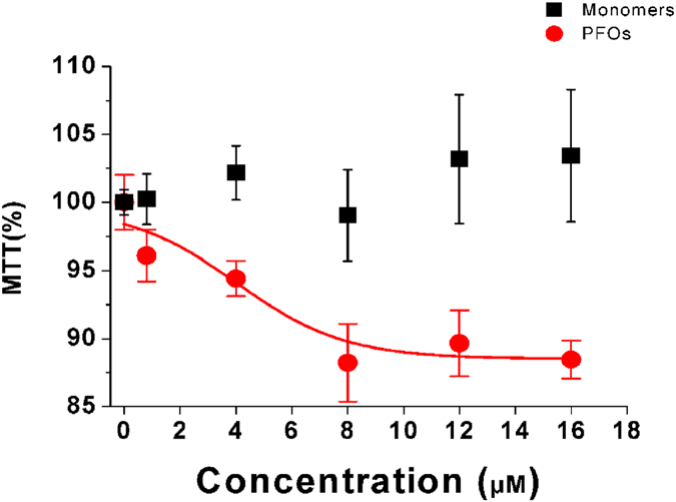
Dose-Response. Dose-Response relationship of the viability induced by sCT PFOs- with respect to Monomers- rich solutions in HT22 DIFF cells.

**Figure 4:**
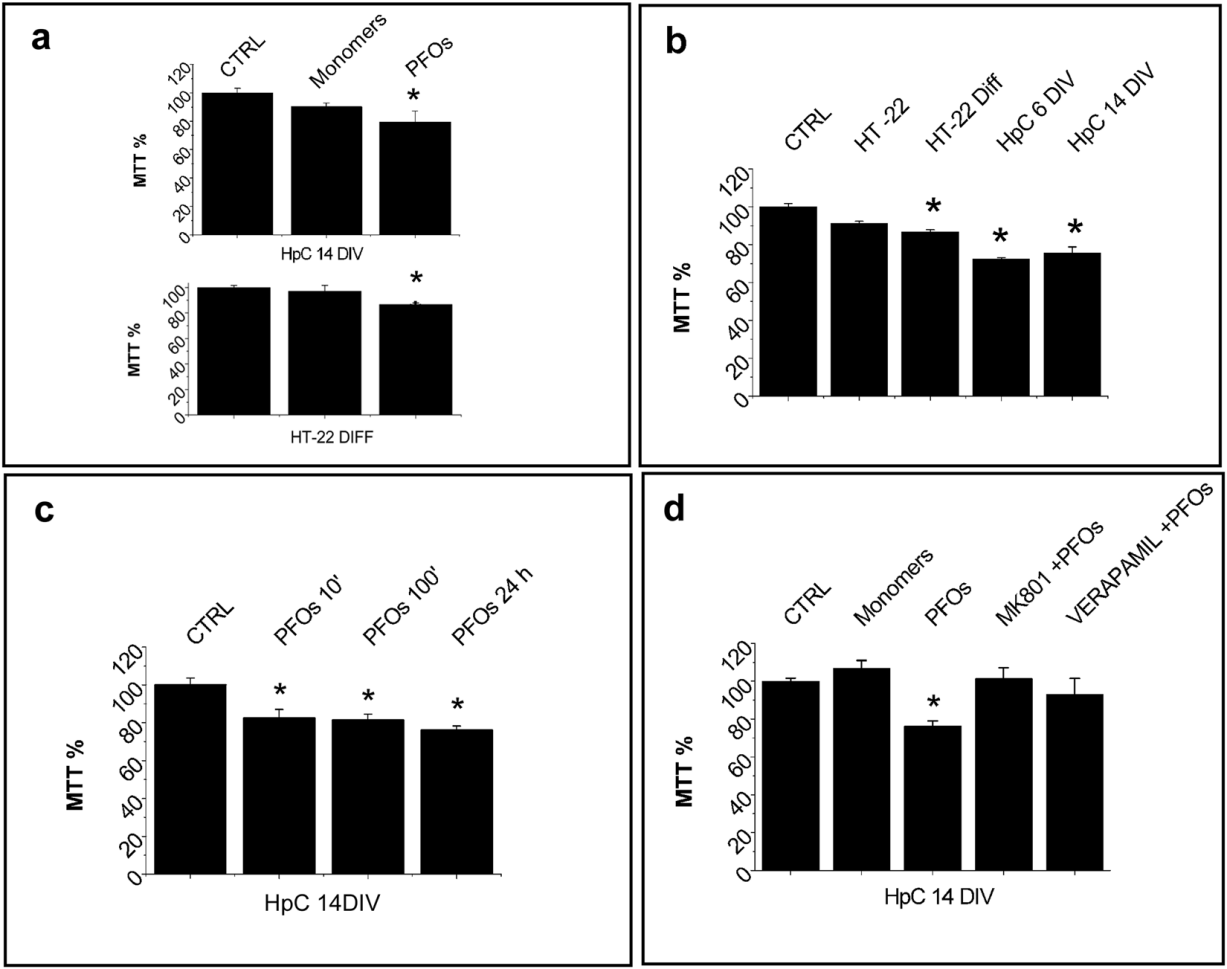
Cell viability results. (**a**) shows that PFOs- but not Monomer-enriched solutions reduced cell viability in both 14 DIV primary hippocampal neurons (CTRL n = 20 wells from N = 4 experiments, Monomers n = 17 wells from N = 3 experiments, PFOs n = 31 wells from N = 3 experiments) and HT22 DIFF cells (CTRL n = 51 wells from N = 9 experiments, Monomers n = 14 wells from N = 4 experiments, PFOs n = 192 wells from N = 38 experiments), after 24 h of incubation. This effect depends from the cell differentiation degree and tends to disappear in undifferentiated HT22 cells (**b**) (CTRL n = 135wells from N = 25 experiments, HT22 n = 194 wells from N = 8 experiments, HT22 DIFF n = 192 wells from N = 38 experiments, HpC 6 DIV n = 31 wells from N = 3 experiments, HpC 14 DIV n = 31 wells from N = 3 experiments). (**c**) Shows that the cell viability was reduced even after only 10 min of incubation with PFOs with 14 DIV primary hippocampal neurons (CTRL, 10 min, 100 min and 24 h n = 10 wells from N = 3 experiments). (**d**) Demonstrates that the Ca^2+^-channel blocker (MK801 and Verapamil) were able to protect against the PFOs neurotoxicity (CTRL n = 15 wells from N = 3 experiments, Monomers n = 12 wells from N = 3 experiments, PFOs n = 40 wells from N = 3 experiments, MK801 + PFOs and Verapamil + PFOs n = 11 wells from N = 3 experiments). CTRL is relative to the untreated specific cell (**a**–**d**) while in (**b**) to cell kind normalized to 100%. With “control” we intend “vehicle-treated” samples.

To clarify if neurotoxicity is correlated to the aggregation state of sCT, we studied the dose-response relationship of PFOs- and Monomers-enriched solutions in HT22 DIFF cells, after 24 hours. Results clearly showed that only PFOs-enriched solutions induced cell viability reduction (about 10%) while Monomers-enriched solutions were totally harmless (Fig. 3**)**. Notably, the same result was obtained with 14 DIV hippocampal neurons (Fig. 4a). Based on these results we decided to perform all viability esperiments at 8 μM concentration.

We noted that cell viability reduction depended from the cell differentiation degree. In fact, as reported Fig. 4a, it was higher in hippocampal neurons (about 25%) with respect to HT22 DIFF. Notably, undifferentiated HT22 cells didn’t reach a significant toxicity (Fig. 4b). Finally, we noted that the loss of viability was not time-dependent, since after 10, 100 minutes and 24 hours of treatment, the same reduction was observed in 14 DIV hippocampal neurons (Fig. 4c).

Thus, we investigated if a correlation exists between the [Ca^2+^]_i_ increase and cell viability. In good agreement with the Ca^2+^-influx experiments, MTT results clearly showed a direct relationship between cell differentiation and/or GM1 expression and PFOs induced neurotoxicity.

As in the case of Ca^2+^-influx, before PFOs administration we pre-treated our cell cultures with MK801 and Verapamil, which block Ca^2+^ channels. In agreement with Ca^2+^-influx results, we found that these blockers fully reverted neurotoxicity. This indicates a direct relationship between neurotoxicity and levels of [Ca^2+^]_i_ reached after 10 minutes and more.

In order to confirm that the reduction of cell viability observed with MTT assay was mainly due to the reduction of neuronal cells instead of glia, we performed immunofluorescence analysis and TUNEL assay of PFOs treated hippocampal cultures. Results (Fig. S3) showed a reduced expression of NeuN positive cells, indicating that the damaged cells were neurons. Moreover, the colocalization of NeuN and TUNEL assay confirmed that PFOs treatment induced apoptosis in neurons.

## Acute PFOs Administration to Brain Slices

### Synaptic plasticity

Over the last decades, the link between oligomers neurotoxicity and synaptic plasticity impairments has been investigated for Aβ^6,30,31,45,48,50,52–56^. As well known, Aβ is an unstable protein and, in agreement with Benilova *et al*., sample changed along different preparation making interpretation and direct comparison of data between different research groups very difficult^5^. At the present, any attempt to test the effects of amyloid native PFOs and Monomers stable during the experiment on synaptic plasticity hasn’t been obtained yet. Herein, we investigated the effects of native samples, as intended in the introduction^3,4^, on synaptic plasticity.

PFOs and Monomers were diluted in carboxygenate Artificial CerebroSpinal Fluid (ACSF) at a final concentration of about 3 μΜ and used to superfuse mouse hippocampal slices.

**Figure 5:**
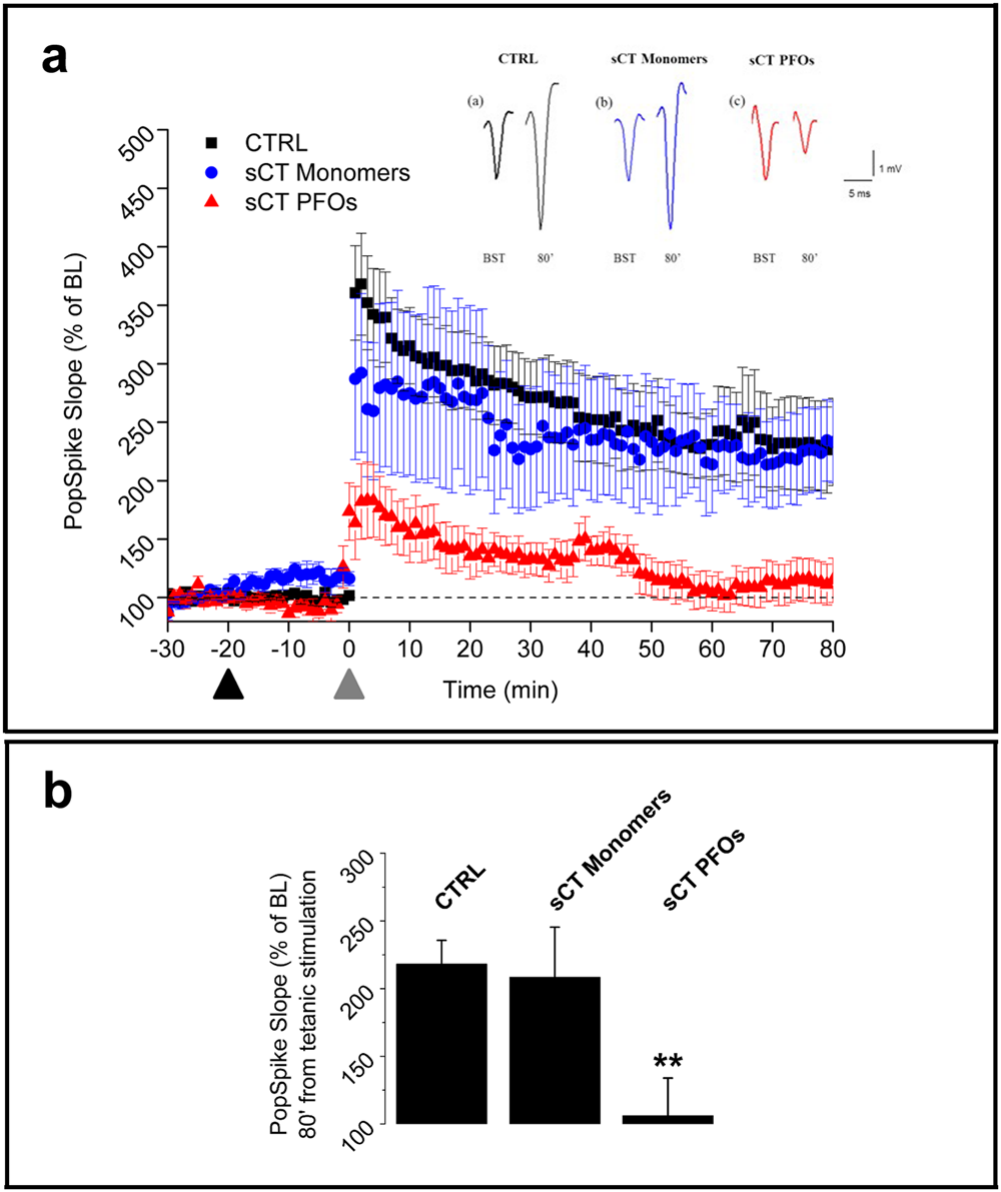
Long-Term Potentiation results. (**a**) shows spectra relative to LTP induced in hippocampal brain slices by sCT PFOs- (n = 6) and Monomer-enriched solutions (n = 6), compared to untreated samples (CTRL) (n = 6). The black arrow represents the sample administration while grey arrow the tetanic stimulation (signals are reported in the inset of a). (**b**) shows the level reached after 80 min from the tetanic stimulation. With “control” we intend “vehicle-treated” samples.

sCT PFOs-enriched samples fully abrogated LTP in hippocampal slices, 80 minutes after the tetanus, while native Monomer-enriched solutions did not affect LTP even when compared to control (Fig. 5). Interestingly, LTP reduction in the case of sCT PFOs was very similar to that reported for Aβ^6^. However, for the first time to our knowledge, we report significant differences between effects induced by sCT native amyloid PFOs- and Monomer-enriched solutions.

### Western blotting and Immunofluorescence of synaptic proteins

In order to evaluate if the altered activity of LTP was correlated with an impairment of synaptic structures^57,58^ and loss of dendritic spines^59^, we studied the expression of a pivotal post-synaptic protein such as PSD-95 and the expression of the pre-synaptic synaptophysin, a component of synaptic vesicles.

**Figure 6:**
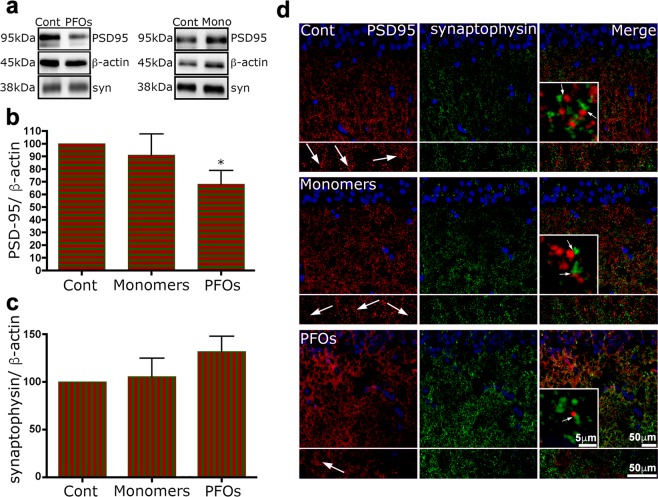
The treatment with sCT oligomers reduced PSD-95 levels in hippocampal slices. (**a**) shows WB analysis of hippocampal slices treated or not with sCT PFOs- or Monomer-enriched solutions. In (**b**,**c**), bar graphs represent mean ± SEM from 4 independent experiments as in a. In b, significantly decreased levels of PSD-95 are observed after treatments with sCT PFOs. In c, WB analysis failed to highlight differences in synaptophysin expression. *p < 0.05 versus control, Mann–Whitney U test. (**d**) After treatments, hippocampal slices were immunolabelled with antibodies anti-PSD-95 (red) or anti-synaptophysin (green) and counterstained with Hoechst 33258. Immunofluorescence analysis confirmed the results obtained with WB analysis. Arrows indicate PSD-95 positive dots; they persist after treatment with Monomer-enriched solutions, but appear reduced in PFOs treated section.

In hippocampal slices incubated with sCT PFOs, WB analysis (Fig. 6a) showed a significant decrease in the expression of PSD-95 with respect to untreated slices (Fig. 6b). Under the same conditions, the levels of synaptophysin remained unmodified (Fig. 6c).

Notably, in agreement with LTP results, our findings showed that the treatment with sCT Monomer-enriched solutions did not modify the levels of these proteins (Fig. 6b,c). The analysis of immunofluorescence confirmed the reduced levels of PSD-95 after treatment with sCT PFOs and the substantial invariance of synaptophysin (Fig. 6d).

## Discussion

Our experimental results demonstrated that sCT was a useful tool to prepare native PFOs- or Monomer-enriched samples to be tested *in vitro* without the need of cross-linking procedure or to apply more sophisticated approaches^9^. Due to its very low aggregation rate, PFOs samples were stable during experiments and rich in aggregates not greater than hexamers. These properties allowed us to overcome difficulties highlighted by Benilova *et al*. in the identification of the amyloid structures responsible for neurotoxicity^5^. Moreover, the sCT PFOs morphology and, more important, the biologic effects they induced were very similar to those reported for toxic Aβ oligomers^3,5^.

**Figure 7:**
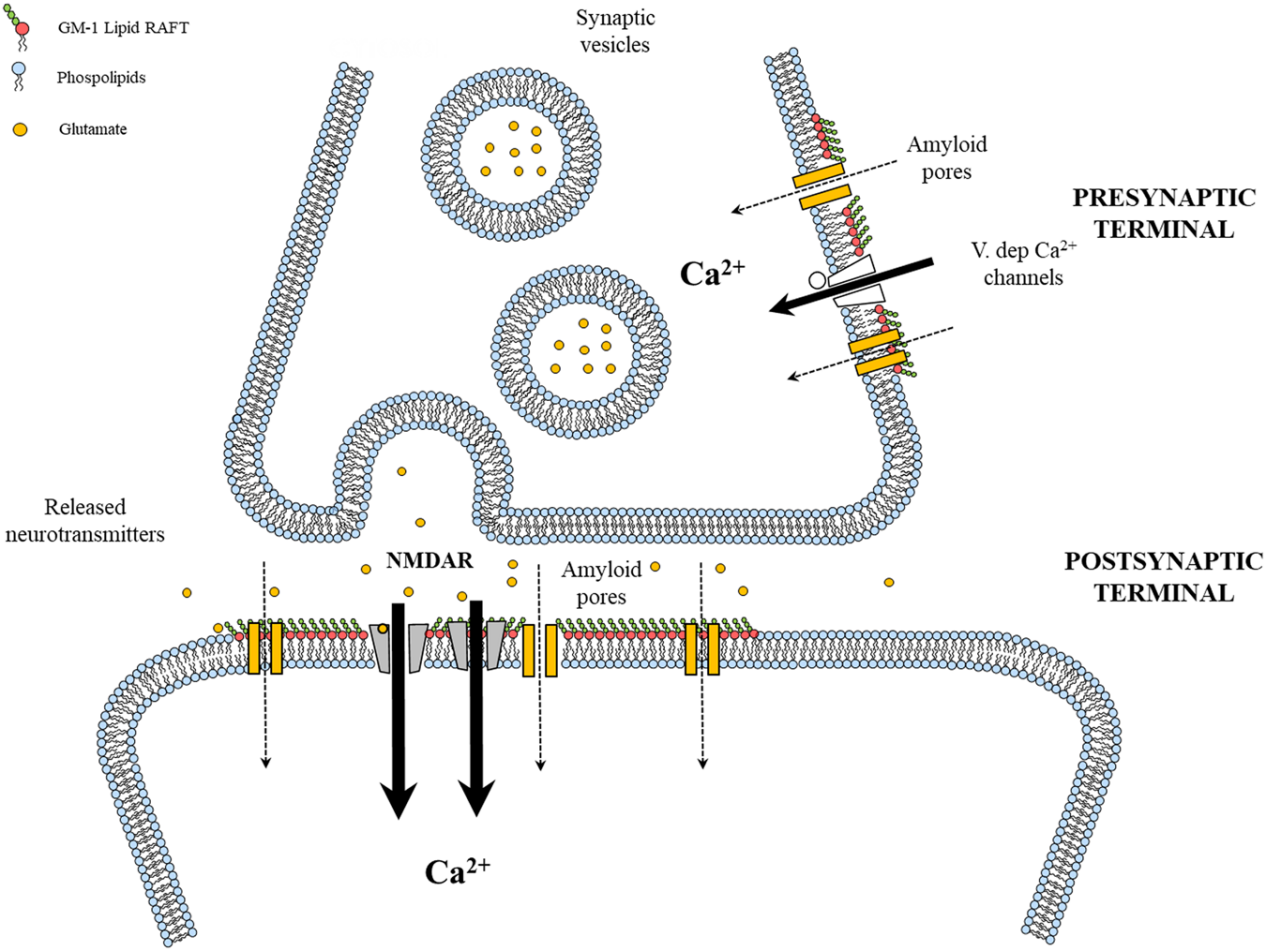
An innovative neurotoxicity mechanism. Amyloid pores formation could represent the initial phenomenon able to trigger the observed subsequent NMDA-mediated abnormal Ca^2+^-influx, leading to the neuronal impairment and damage.

We demonstrated that native soluble sCT PFOs-rich samples, composed of tetramers, pentamers and hexamers but not species of higher molecular weights, were able to induce sustained Ca^2+^-influx in mature mouse primary hippocampal neurons (14 DIV) (Fig. 2a). Interestingly, when NMDARs were blocked by MK801 a small and transitory but detectable Ca^2+^-influx was still observed (Fig. 2c). Notably, in a previous work we observed the same influx by treating with sCT oligomers rat mature primary neurons pre-treated with MK801 (see Fig. 7 therein)^14^. This suggests that the main part of the observed Ca^2+^-influx in mature neurons was due to NMDAR activation^35^ but that a residual NMDAR-independent component exists.

In our opinion, this component could be ascribed to the formation of amyloid pores. We base this hypothesis on our data relative to undifferentiated HT22 cells (Fig. 2b). As shown by He *et al*., these cells do not express cholinergic and glutamatergic receptors, and in particular NMDAR^51^. We carefully checked this fact by treating cells with NMDA and we did not observe any Ca^2+^-influx (Fig. S2). PFOs were able to induce a Ca^2+^-influx transitory peak even in undifferentiated HT22, where NMDAR was absent. Thus, we hypothesize that the influx can be due to the formation of amyloid pores.

We note that several groups proposed that the presence of GM1 is necessary for pores formation^13,54–56^. Here we report that undifferentiated HT22 cells expressed low but detectable levels of GM1 (Fig. 2d). Thus, we hypothesize that in these cells few amyloid pores can be formed, with the result to create the observed weak and transitory Ca^2+^-influx, insufficient to affect cells viability (Fig. 4c). Notably, we observed the same peak in 14 DIV primary neurons pre-treated with MK801, where NMDARs were blocked (Fig. 4d).

It is interesting to note what happened working with HT22 DIFF cells that express a higher level of GM1 (Fig. 2d) and, at the same time, express glutamatergic receptors^51^ (Fig. S2). The Ca^2+^-influx was more than doubled in intensity and remained sustained beyond 10 minutes (Fig. 2b) and was sufficient to induce a significant (about 10%) cell viability decrease (Fig. 4b).

We speculate that the formation of more amyloid pores, determined by the higher GM1 content, induced a stronger Ca^2+^-influx leading to the activation of the NMDARs. This phenomenon can lead, in our opinion, to the observed cell death through mitochondrial dysfunction and oxidative damage, as proposed by Angelova and Abramov^37^.

In good agreement, when primary neurons (6 DIV, 14 DIV) were studied, the high GM1 content (Fig. 4b) leads to the formation of many amyloid pores, enough to boost up the abnormal activation of the NMDARs described before in differentiated HT22 cells. In 6 DIV and more in 14 DIV, the Ca^2+^-influx curves were very similar to that observed for HT22 DIFF but more than doubled (Fig. 2b) and a cell viability reduction of about 25% (Fig. 4b).

The crucial role played by GM1 in the formation of amyloid pores by PFOs, has been recently highlighted by Hong *et al*. for Aβ^24^. Furthermore, in a previous paper we showed that masking GM1 in rat primary hippocampal neurons totally prevents Ca^2+^-influx and toxicity induced by sCT oligomers^14^.

However, the results we are presenting now clearly indicate that the formation of amyloid pores alone is not enough to explain neurotoxixcity and that NMDARs must be involved, as suggested by the protection exerted by MK801. Finally, we note that none of the effects described before was induced by sCT Monomer-enriched samples, containing a minority of dimers and trimers. As proposed by Angelova and Abramov, this can be due to the inability of Monomers to form stable amyloid pores and to switch on the pathological Ca^2+^-influx and the consequent neurotoxicity^37^.

For what concerns LTP experiments, we showed that sCT PFOs abrogated synaptic plasticity after 100 minutes of treatment while, in good agreement with neurotoxicity results described before, Monomer-enriched solutions were totally ineffective (Fig. 5). Similar results have been reported for aggregates of others amyloid proteins^5,6,30,50,53^. However, in our knowledge, the direct comparison between the effects induced by PFOs and Monomers has been never investigated for sCT.

Interestingly, we provide evidences that cells were damaged after 10 minutes as after 24 hours of PFOs treatment (Fig. 4c). This suggests that, in the LTP experiments, also neurons can be damaged after 10 minutes of PFOs treatment. However, neurons were still alive since responded to the tetanic stimulation producing population spikes. Angelova and Abramov have shown that ROS production in primary neuronal culture is significantly higher after 8–10 minutes of oligomer exposure, compared to Monomer treatment^37^.

Thus, we interpret our LTP observations as a consequence of the Ca^2+^-influx mechanism proposed before. In our hypothesis, once Ca^2+^ ions were allowed to flow into the cells, the following membrane depolarization with the consequent abnormal Ca^2+^ conductance, impaired synaptic transmission and produced vesicle depletion resulting in synapse silencing^31^. This interpretation is supported by our results relative to the PSD-95 expression in brain slices used in the LTP experiments. Notably, we found reduced levels of PSD-95 and the invariance of synaptophysin expression, induced by sCT PFOs- but not by Monomer-enriched solutions (Fig. 6a,b).

According to the scenario depicted before, even if data reported here are not sufficient to demonstrate the formation of amyloid pores in a definitive manner, we hypothesize (Fig. 7) that their formation could represent the initial phenomenon able to trigger the observed subsequent NMDA-mediated abnormal Ca^2+^-influx, leading to the neuronal impairment and damage.

Thus, if a common amyloid-related excitotoxicity mechanism exists, as proposed by Glabe^2^, our results can be important in the design of novel therapeutic approaches that should follow three main routes: i) to avoid the PFOs formation before they might interact with neuronal membranes; ii) to avoid the binding between amyloid PFOs and GM1; iii) to block amyloid pores once they were formed, before the NMDA-mediated Ca^2+^-influx is activated.

## Methods

### sCT samples preparation by Size Exclusion Chromatography (SEC)

Monomer-enriched solutions at 1 mM sCT was prepared by dissolving sCT lyophilized powder (European Pharmacopoeia, EDQM, France) in desalted water. In order to limiting the aggregation process, the solution was rapidly frozen and stored at −80 °C. On the contrary, aggregated, native sCT solution was prepared incubating 2 mg of sCT powder in 5 mM, phosphate buffer (PB: PB 5 mM, pH 7.4), at room temperature overnight. The aggregated and native solution was then loaded in the SEC column in order to purify oligomeric species enriched fractions^3^. Briefly samples were loaded in Sephadex G50-SEC column (GE HEALTHCARE, Milano, Italy- height: 500 mm, section: 20 mm). Column, maintained at 4 °C was pre-equilibrated at the same ionic strength of the samples and calibrated with a solution containing standards: BSA 1 mg (66 kDa), Cytochrome c 1 mg (12.4 kDa-Combithek Boehringer, Mannheim, Germany), Aprotinin 1 mg (6.5 KDa) and Somatostatin 1 mg (1.63 KDa) suspended in 5 mM PB buffer pH 7.4, and centrifuged at 15700 g × 10 min. Monomeric or aggregated sCT native solutions (0.5 mL aliquots), prepared as descripted above, were eluted in the column monitoring absorption at 280 nm by a variable wavelength UV detector (BIORAD Econo UV monitor, Hercules, CA). Fractions collected (Gilson FC 203B, 1.4 mL/fraction) were administered directly in cell cultures, to test their effects on cellular viability and calcium imaging and in hippocampus slices to evaluate LTP. The final concentrations of sCT fractions used in this experiments was 8 μM.

### Photo-induced Cross-linking of Unmodified Proteins (PICUP) and tricine SDS-PAGE Gel characterization

Aiming to obtain an electrophoretic characterization (tricine/SDS-PAGE) of the fraction eluted, samples were stabilized in order to prevent oligomers misfolding in a reducing environment. We therefore treated them for PICUP. The original protocol was adapted by Diociauti *et al*.^3^. Briefly, for each sample, we prepared a 20 μl volume containing 80 μM Oligomers, 50 μM Tris (2,2 bipyridyl) dichlororuthenium (II) hexahydrate) and 1 mM ammonium persulfate (SIGMA). Cross-linking reaction occurred irradiating samples for 2 s with a 100 W white lamp in a dark room, reaction was quickly quenched adding 20 μl of reducing sample buffer containing 5% β-MeOH and boiled for 5 minutes. Samples were finally analysed by tricine/SDS-PAGE. Separating gel: 10% Acrylamide/Bis (32:1) (ICN Biomedicals, Inc., Aurora, Ohio USA/Fluka, Buchs, CH); spacer gel 6.5% Acrylamide/Bis and stacking gel: 2.5% Acrylamide/Bis. For each lane were run 40 μl of the sample buffer, or 8 μl of molecular weight markers (Color Marker Ultralow Range -SIGMA, cat n° C6210-1VL). Then gels were stained by silver procedure. Finally, gel band densitometry has been performed using the imaging freeware ImageJ. For each lane, grey-scale optical density profile has been obtained, including molecular weight markers (1.3 KDa, 3.5 KDa, 6.5 KDa, 14.4 KDa, 17.0 KDa, 26.6 KDa). Peaks corresponding to sCT prefibrillar oligomers: Monomers, dimers, trimers-tetramers, pentamers and hexamers has been individuated according to the molecular weight marker profile. Gel densitometry was used to compute two estimators, named *PFOs % index*and *Average MW index*, taking in account for the oligomers species that prevalently populated the fractions. The values were used to evaluate statistical differences between sCT monomeric and aggregated samples.

### Energy Filtered-Transmission Electron Microscopy (EF-TEM)

We used a Transmission Electron Microscope model TECNAI 12 G2 Twin (FEI Company, Hillsboro, OR, USA) equipped with a thermionic gun (single-crystal lanthanum hexaboride) and energy dispersive X-ray spectrometer (model Genesis 4000, EDAX Inc., Mahwah, NY, USA) and post-column electron energy filter (Bio filter, GATAN Inc., Pleasanton, CA, USA). The energy filtered images were acquired by the use of a slow-scan CCD camera (model 794 IF, GATAN Inc., Pleasanton, CA, USA). Conventional imaging was performed in energy-filtered image mode configuration at electron energy of 120 keV, with a collection angle of about 20 mrad. To enhance image contrast and resolution, chromatic aberrations were reduced by collecting only elastic electrons (Δ*E* = 0)^60^.

### Cell cultures

HT22 cells were developed from their analogous HT4 cells, immortalized from primary mouse hippocampal neurons. If grown without establishing synaptic connections, both HT22 and HT4 hippocampal cells are able to develop LTP, in terms of neurotransmitter release^29^. HT22 neuronal cell line in proliferating conditions, doesn’t express cholinergic and glutamatergic receptors, although HT22 cells can also be differentiated in a selective medium, changing their morphology and inducing expression of cholinergic markers like: choline acetyl transferase (ChAT), vesicular acetylcholine transporter (VAChT), high affinity choline transporter (HACT), muscarinic M1 and M2 subunit of Ach- receptors. Moreover, they become susceptible to glutamate excitotoxicity^51,61^ _ENREF_3 that is mainly mediated by NMDARs^62^ via Ca^2+^-influx^63^, but also by several second messengers like nitric oxide^64^, calpain-1/poly-(ADPribose) polymerase1/apoptosis inducing factor^65^, free radicals and mitochondria^66^. NMDAR antagonists, such as Dizocilpine (MK801), can effectively prevent glutamate-induced excitotoxicity^51^.

HT22 cells were maintained at 37 °C, 10%, CO_2_ in Dulbecco’s modified Eagle’s medium (DMEM, Sigma Aldrich–D6546) supplemented with 10% heat-inactivated FBS, and kept at less than 50% of confluence. Differentiation was carried out in NeuroBasal medium (NBM, Gibco, 21103-49) containing N2 supplement (Gibco-17502048), at least for 24–48 hours before use.

Primary hippocampal co-cultures (neurons and glia) have been prepared from postnatal day 2-mouse brain. All experimental procedures were carried out according to the Italian law and to “Ethical guidelines for scientific experimentation on animals”. Experimental protocol was approved by the Italian Ministry of Public Health (authorization n. 86/2018-PR), and was in accordance with guidelines of the European Union Council Directive (86/609/European Economic Community). After dissection, hippocampi were incubated 15 minutes at 37 °C, with 0.25% trypsin (Gibco, 15090-046) and then dissociated in NeuroBasal medium (Gibco, 21103-49) containing 10% heat-inactivated FBS, 50 µg/mL gentamicin (Gibco, 15750-037), 1x Glutamax (Gibco, 35050-038). Cells were seeded on 48-wells plates for MTT assay or on sterile glass coverslips (diameter 12 mm), previously coated with 1x poly-L-lysine (Sigma-Adrich), in 24-wells plates, for Fura2-AM experiments or GM1 evaluation. After plating cell cultures were rapidly stored in the cell incubator (5% CO_2_, 37 °C). After 2 hours, cell culture medium was replaced with 700 μl/well of Neurobasal medium containing 1X B27 supplement (Gibco, 17504-044) instead of FBS. Hippocampal co-cultures were used at 6 DIV (not fully mature culture) or 14 DIV (fully mature culture).

### Fura-2AM Ca^2+^ imaging recordings in cell cultures

For evaluation of the intracellular Ca^2+^ ([Ca^2+^]_i_), 1 mM Fura-2 Acetoxy Methyl ester (Fura-2AM, Thermo Fisher Scientific - F1221) stock solution was prepared dissolving Fura-2AM lyophilized salt in 75% DMSO, 25% pluronic acid (Thermo Fisher Scientific, F-127), sonicated for 5 minutes in darkness, at RT. 5 μM Fura-2AM solution was diluted from stock solution in fresh Ringer extracellular solution (NaCl 125 mM, KCl 1 mM, MgCl_2_ 1.5 mM, CaCl 2 mM, HEPES 20 mM, D-Glucose 8 mM, 300 mOsm, pH 7.4 with NaOH). Cell-seeded coverslips were incubated with Fura-2AM working solution for 50 minutes at 37 °C, 5% CO_2_ in darkness. After three washes in Ringer solution, coverslips were rapidly placed in the cell bath with fresh Ringer solution on the microscope stage, for Ca^2+^ imaging recordings. All recordings were performed in dark conditions. After 2 minutes of adaptation (baseline), samples from different native sCTs fractions were used to treat the cells, at a final concentration of 8 µM. Fluorimetric recordings with Fura-2AM were performed along the experiment to obtain ratiometric measurements of the [Ca^2+^]_i_ that is the ratio between the emission intensities measured at 510 nm, stimulated at 340 nm and 380 nm^67^. One acquisition was done each 6 second, along 15 minutes, on a region of interest in the cellular bodies. Ratios and 340 nm and 380 nm background signals, from time laps sequence images were obtained by the imaging freeware ImageJ. Data were plotted by the software: Microcal Origin 8.

### MTT and TUNEL assay in cell cultures

Cell viability was evaluated by the 3-(4,5-dimethylthiazol-2-yl)-2,5-diphenyltetrazolium bromide (MTT) assay; the MTT assay has been widely used to assess cell viability and is based on the ability of viable cells to reduce MTT, giving rise to an insoluble purple formazan salt. Briefly, the cultures were incubated for 20 minutes at 37 °C with 0.5 mg/ml MTT in Hank’s balanced salt solution (Life Technologies). The reaction product was dissolved in dimethyl sulphoxide. The spectral photometric absorbance of the samples was determined at a wavelength of 540 nm. The amount of MTT conversion was evaluated as a percentage of the absorbance measured in treated cells relative to the absorbance of control cells. After fixation in 4% paraformaldehyde in PBS, 0.12 M in sucrose, apoptosis was evaluated in mixed hippocampal cultures by the terminal transferase-mediated dUTP-biotin nick end-labeling (TUNEL) assay (DeadEnd kit, Promega, Madison, WI).

###  Long-Term Potentiation (LTP) in mouse hippocampal slices

Wild type BALB/c mice aged 6 to 9 weeks were used in accordance with guidelines and regulations of the European Union Council Directive (86/609/European Economic Community). All the experimental protocols were approved by the Italian Ministry of Public Health (authorization n. 86/2018-PR). Under anesthesia with halothane (2-Brom-2-chlor-1,1,1-trifluor-ethan), the animals were decapitated and brains were quickly removed and placed in cold, oxygenated artificial cerebral spinal fluid (ACSF), whose composition in mM was: NaCl 124, KCl 2, KH_2_PO_4_ 1,25, MgSO_4_ 2, CaCl_2_ 2, NaHCO_3_ 26, and Glucose 10. The hippocampal slices were prepared according to conventional procedures^68^. The hippocampus was rapidly dissected and slices (450 µm thick) were cut transversely by a chopper (McIlwain Tissue Chopper) and transferred into an interface tissue chamber constantly perfused by a flow of 1.2 mL/min of ACSF and humidified gas (95% O_2_ – 5% CO_2_) at 32–34 °C (pH 7.4). According to the original protocol^69^, extracellular recordings of the population spikes (PSs) were made in the stratum pyramidale of the CA1 subfield using glass microelectrodes filled with 2 M NaCl (resistance 5–10 MΩ). Orthodromic stimuli (10–500 mA, 20–90 ms, 0,1 Hz) were delivered through a platinum electrode placed in the Schaffer collateral commissural pathways in the stratum radiatum. The test stimulus intensity of 50 ms square pulses was adjusted to elicit a PS of 2–3 mV at 0,03 Hz. Each minute, a trace was calculated as the average of six recordings every 10 seconds. After recording stable signals (20–30 minutes), the hippocampal slices were treated with Monomer- or PFOs-enriched solutions of sCT, in order to assay their effects on synaptic plasticity. PFOs and Monomers were diluted in carboxygenate ACSF at a final concentration of about 2 μM: the slices where then superfused.

After 20 minutes from administration of sCT, a tetanic stimulation (100 Hz, 1 s) was delivered to induce LTP at the same stimulus intensity used for the baseline responses. Field potentials were fed to a computer interface (Digidata 1440 A, Axon Instruments, Foster City, CA) for subsequent analysis with the software PCLAMP10 (Axon Instruments).

### Western blot (WB) analysis

Hippocampal 450 μm-thick transversely coronal slices were homogenized in extraction buffer (25 mM Tris–HCl, pH 7.4, 150 mM NaCl, 1% Triton X-100, 0.1% SDS, 1% sodium deoxycholate, 1 mM sodium orthovanadate, 1 mM sodium fluoride, 1 mM PMSF, and a protease inhibitor cocktail) on ice for 30 minutes and centrifuged at 100.000 × g for 1 h, at 4 °C. The protein concentration was determined using the Micro BCA Protein Assay Kit (Pierce, Rockford, IL, USA). Proteins (30 µg) were separated on 12% SDS-PAGE and transferred to nitrocellulose membranes at 35 V overnight. The membranes were blocked at room temperature in 3% BSA and incubated overnight at 4 °C with the following primary antibodies: mouse monoclonal anti-PSD-95, rabbit polyclonal anti-synaptophysin (home-made) and, as a control for protein loading, mouse monoclonal anti-β-actin (Santa Cruz). The membranes were washed and incubated with the appropriate peroxidase-labelled secondary antibody (Bio-Rad, Hercules, CA, USA) for 1 h at room temperature. After extensive washes in TTBS (20 mM Tris–HCl, pH 7.4, 0.15 M NaCl, 0.1% Tween 20), the immunoreactive bands were detected by enhanced chemiluminescence coupled to peroxidase activity (Santa Cruz Biotech) and imaged with a ChemiDoc XRS system (Bio-Rad Laboratories Inc.).

### Immunofluorescence

Hippocampal 450 μm-thick transversely coronal slices were treated or not with sCT PFOs- or Monomer-enriched solutions for 100 min. After treatment, the slices were fixed over night with 4% paraformaldehyde in PBS, 0.12 M in sucrose. After fixation, the samples were rinsed three times in PBS with 5% sucrose and 0.15 mM CaCl_2_ and left overnight in sucrose buffer (PBS with 30% sucrose and 0.15 mM CaCl_2_). Samples were then embedded in Tissue Freezing Medium (Jung, Germany), frozen at −30 °C in isopentane and stored at −80 °C. Sections (8 μm) were cut at a Leica CM 1860 UV cryostat and labelled with primary antibodies overnight at 4 °C. The following primary antibodies were used: monoclonal anti-NeuN (Millipore, USA) rabbit anti-PSD-95 (Cell Signaling Technology, Danvers, MA) and monoclonal anti-synaptophysin (BD Transduction Laboratories, Franklin Lakes, NJ). Primary antibodies were revealed with secondary antibodies coupled to Alexa Fluor® 488 and Alexa Fluor® 546 (Invitrogen), diluted 1:250 PBS (45 min, 37 °C). Sections were counterstained with Hoechst 33258; the dye, which binds specifically to A–T base regions in DNA and emits blue immunofluorescence at 350 nm, was administered at 1 ng/ml for 1 minute. Sections were observed at an Eclipse 80i Nikon Fluorescence Microscope (Nikon Instruments, Amsterdam, The Netherlands), equipped with a Video Confocal (ViCo) system.

### GM1 expression

To analyse GM1 localization in HT22 cells and hippocampal neurons we used Alexa Fluor 488–conjugated cholera toxin-β (CT β, Molecular Probes, Eugene, OR) (10 μg/mL). After fixation in 4% paraformaldehyde in PBS, 0.12 M in sucrose, cultures were stained with CTβ at room temperature for 30 minutes. To highlight nuclei, cultures were counterstained with Hoechst 33258. Cultures were observed at an Eclipse 80i Nikon Fluorescence Microscope (Nikon Instruments, Amsterdam, The Netherlands), equipped with a Video Confocal (ViCo) system.

### Statistical analysis

#### Average Molecular Weight and PFOs % indexes

We computed two estimators from gel densitometry, named:

$$Average\,MW\,index\,(AMWi-kDa)=\sum _{i=1}^{n}Ai\cdot m{w}_{i}$$

(A_i_: Area under the i-meric species band peak)

and

$$PFOs\, \% \,index=(\frac{1}{{A}_{t}}\cdot \sum _{i=1}^{n}{A}_{Te+Tr+P+E})\cdot 100$$

(A_Te+Tr+P+E_: Area under the trimeric, tetrameric, pentameric and hexameric species band peaks, A_t_: total area under the gel densitometry lane).

 A non-parametric test was computed to assess statistical significance of the  index differences, for Monomer- and PFOs-enriched samples, with a confidence level of 95%, under the null hypothesis *H*_*0*_: *AMWi*_*Monomers*_ = *AMWi*_*samples*_ or *PFOs%*_*Monomers*_ = *PFOs%*_*samples*_.

### Intracellular Ca^2+^

Peaks and end-time values of the [Ca^2+^]_i_, were calculated over the experiments and expressed as a percentage of the Baseline-Corrected-Ratio values. Statistical significance of the [Ca^2+^]_i_ differences, between Monomers *vs* PFOs treated neurons was assessed under the null hypothesis: *H*_*0a*_ ([*Ca*^*2+*^]_*i*_) _*Monomers*_ = ([*Ca*^*2+*^]_*i*_) _*PFO*_, by means of T-test, with a confidence level of 95% and 99%. A multiple comparison in the effects of PFOs administration upon drugs pre-treatments with MK801 and Verapamil or in the effects of PFOs administration on cells in different maturating conditions (undifferentiated HT22, differentiated HT22, HpC neurons 6 DIV, and HpC neurons 14 DIV), was assessed respectively by means of one way ANOVA, followed by Dunnett post-tests or for multiple comparison by Bonferroni correction.

### MTT

Cellular viability estimations for each experimental condition were obtained in quadruplicate, from the MTT assay. Data were normalized respect to controls. A multiple comparison in cellular viability was obtained with respect to controls for all experimental conditions, by means of ANOVA followed by Dunnett test, with a confidence level of 95% and 99%.

### LTP

The maximal slopes of the descending and ascending branches of the PS were averaged in module and plotted against the corresponding experimental time. For each trace, raw data were normalized as a percentage of the baseline PS slope, before test substances administration. Statistical significance in differences for all experimental conditions, was assessed by means of an ANOVA followed by Bonferroni correction, with respect to the basal transmission and to the end-time values, (80 minutes after the tetanic stimulation), with a confidence of 95% and 99%.

## Supplementary information

Supplementary Materials
