# Supplementary material for: Calcitonin native prefibrillar oligomers but not monomers induce membrane damage that triggers NMDA-mediated Ca2+-influx, LTP impairment and neurotoxicity
Source: Sci Rep. 2019 Mar 26;9:5144. doi: 10.1038/s41598-019-41462-0 (PMC6435710; doi:10.1038/s41598-019-41462-0)
Supplement: Supplementary file 1 — Supplementary Materials [file 41598_2019_41462_MOESM1_ESM.pdf]

Supplementary materials

Calcitonin native prefibrillar oligomers but not monomers induce membrane damage that triggers NMDA-mediated Ca^{2+}-influx, LTP impairment and neurotoxicity

Marcello Belfiore[1], Ida Cariati[2], Andrea Matteucci[3], Lucia Gaddini[3], Gianfranco Macchia[4], Raoul Fioravanti[1,5], Claudio Frank[1], Virginia Tancredi[2], Giovanna D'Arcangelo[2,#] and Marco Diociaiuti[1,#,*].

[1]National Center for Rare Diseases, Istituto Superiore di Sanità, Rome, Italy; [2]Department of Systems Medicine, University of Rome Tor Vergata, Rome, Italy; [3]National Center for drug research and evalutation, Istituto Superiore di Sanità, Rome, Italy; [4]Core Facilities Service, Istituto Superiore di Sanità, Rome, Italy; [5]Chemistry Department, University "Sapienza", Rome, Italy.

*Corresponding author: marco.diociaiuti@iss.it
[#] this have to be considered as Last Authors

GEL Analysis

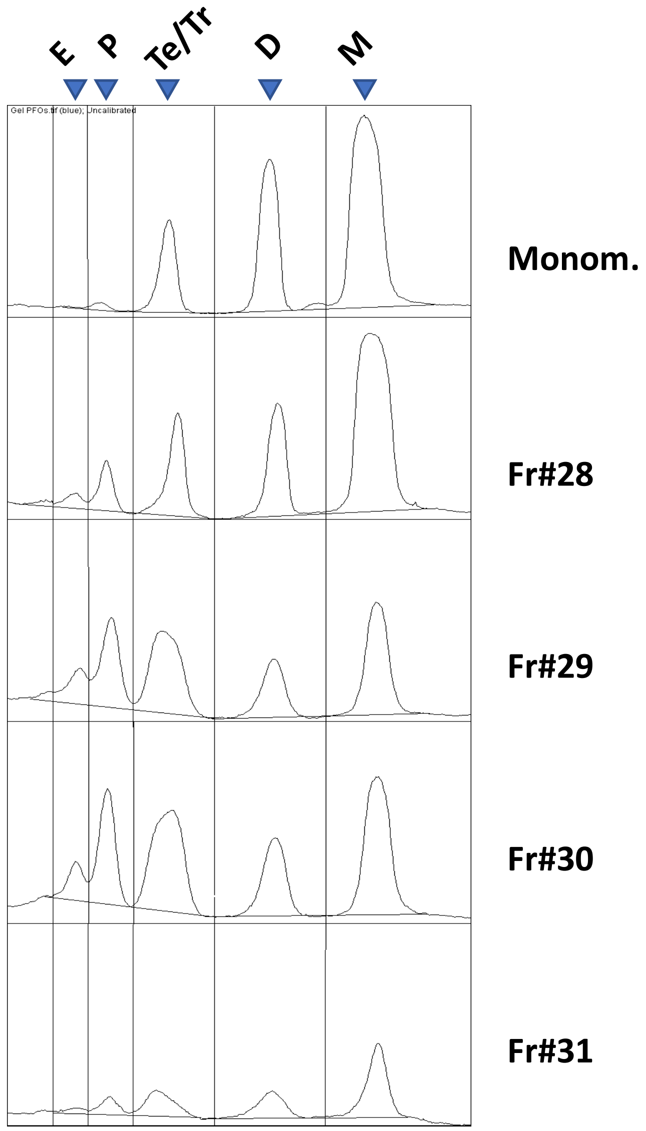

Figure S1 - We report below the gel lane densitometry, obtained in ImageJ from figure1 panel B. for each gel lane, band peaks are depicted. From right to left to right: (E) hexamers, (P) pentamers, (Te) tetramers /(Tr) trimers (which are not fully resolved), (D) dimers and (M) monomers. We are in the linear range.

The areas under the curves obtained in ImageJ for each lane of figure 1 B are reported in the table below. Areas were used to compute two estimators named Average Molecular weight index and (Te/Tr+P+E) PFOs %.

**MW(Kda)**						
		**MONOMERS**	**FR.28**	**FR.29**	**FR.30**	**FR.31**
**20,6**	Esamers	22,536	740,163	1828,355	1685,062	308,849
**17,2**	Pentamers	328,213	1885,184	4660,79	5099,962	898,062
**‡12**	Tetramers/Trimers	4129,912	4630,225	6919,125	8523,539	1933,619
**6,8**	Dimers	7519,912	5516,983	3582,104	4828,811	1994,74
**3,4**	Monomers	15215,903	15417,439	6834,468	9432,418	4134,933
		**PFOs%**	**PFOs%**	**PFOs%**	**PFOs%**	**PFOs%**
		16,5	25,7	56,3	51,8	33,9
		**AMW$_I$**	**AMW$_I$**	**AMW$_I$**	**AMW$_I$**	**AMW$_I$**
		5,82	6,85	10,42	9,79	7,83

‡ the average of the molecular weight for trimmers (10,2 KDa) and Tetramers (13,6KDa)

NMDA effect on HT 22 cells

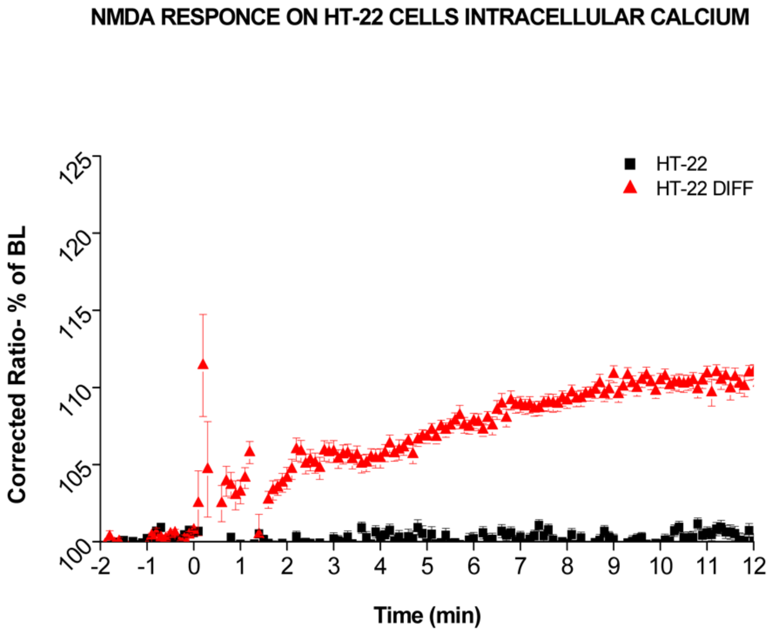

Figure S2 – Ca^{2+}-influx induced by NMDA in HT 22 and HT 22 DIFF cells

Immunofluorescence analysis and TUNEL assay

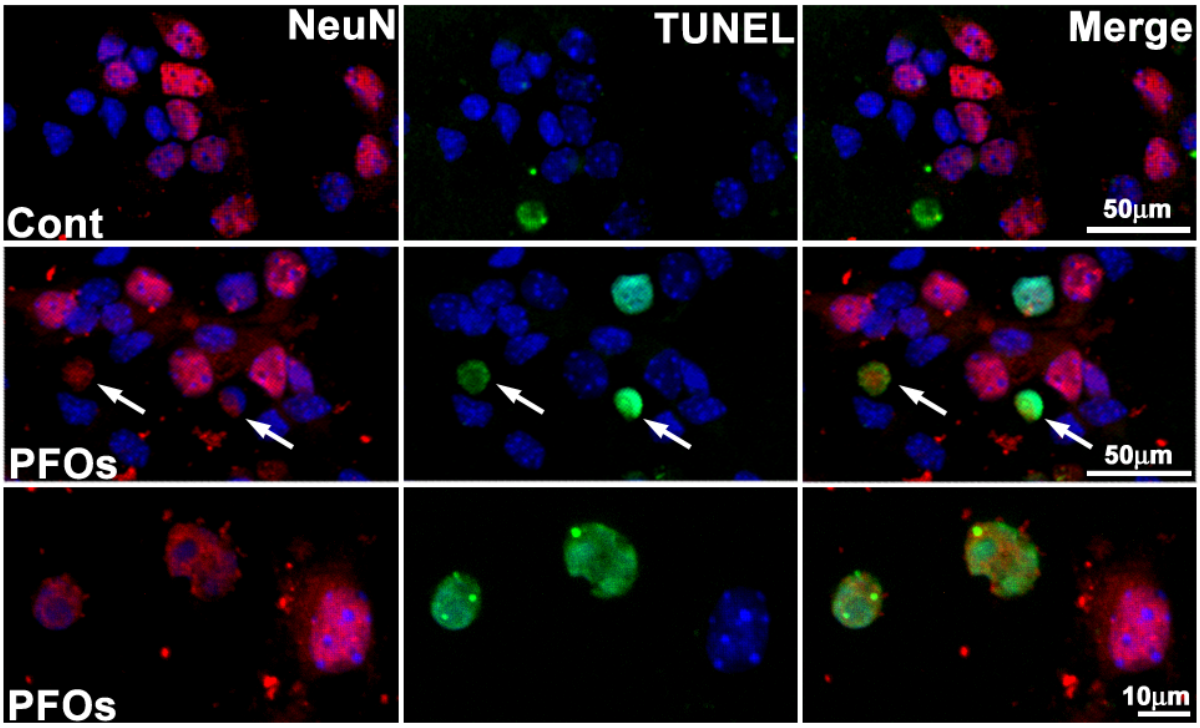

Figure S3 - The treatment with sCT oligomers induces apoptosis in mixed hippocampal cultures. Hippocampal cultures at DIV 14 were immunolabelled with antibody anti-NeuN (red) to highlight neuronal cell; apoptosis was evaluated by the TUNEL assay (green). After treatment with PFOs we observed an increase in apoptotic nuclei that appear to colocalize with NeuN (arrows). The second row is at higher magnification. Nuclei were counterstained with Hoechst 33258 (blue).
